# Development of a Multifunctional Chitosan-Based Composite Film from Crab Shell (*Portunus segnis*) and Algae (*Ulva lactuca*) with Enhanced Antioxidant and Antimicrobial Properties for Active Food Packaging

**DOI:** 10.3390/foods14010053

**Published:** 2024-12-27

**Authors:** Imen Zaghbib, Johar Amin Ahmed Abdullah, Alberto Romero

**Affiliations:** 1 Research Laboratory “Technological Innovation and Food Security-LR22 AGR01”, Higher Institute of Food Industries of Tunisia (ESIAT), University of Carthage, Tunis 1002, Tunisia; zaghbibimen@gmail.com; 2 Department of Chemical Engineering, Faculty of Chemistry, Universidad de Sevilla, 41012 Seville, Spain

**Keywords:** Chitosan, *Ulva lactuca*, composite film, physicochemical properties, biological activities

## Abstract

Eco-friendly, bioactive and edible films from renewable resources are increasingly regarded as viable replacements for petroleum-based packaging. This study investigates the application of *Ulva lactuca* macroalgae powder (ULP) as an active additive in crab (*Portunus segnis*) chitosan-based films for natural food packaging. Films with ULP concentrations of 0.5, 1.5, and 2.5% were prepared using a solvent-casting method with glycerol as a plasticizer. Their physicochemical, mechanical, functional, and biological properties were evaluated comprehensively. Fourier-transform infrared spectroscopy revealed intermolecular interactions between ULP’s polyphenolic compounds and the chitosan matrix, enhancing the films’ structural integrities. ULP’s incorporation reduced the moisture content, water solubility, lightness (L*), redness (a*), and whiteness index values while significantly (*p* < 0.05) increasing the yellowness (b*), total color difference (ΔE), yellowness index (YI), tensile strength (TS), and elongation at break (EB). The antioxidant activity improved in a concentration-dependent manner, as evidenced by the high free-radical scavenging capacity. Moreover, antimicrobial tests showed significant inhibitory effects against pathogenic strains. Biodegradability tests confirmed that the films decomposed entirely within 12 days under soil burial conditions, reinforcing their environmental compatibility. These results highlight the multifunctional potential of chitosan–ULP composite films, combining enhanced mechanical properties, bioactivity, and sustainability. By utilizing renewable and biodegradable materials, this work contributes to reducing waste and promoting resource efficiency, aligning with the principles of a circular economy and environmental preservation.

## 1. Introduction

In recent years, considerable research has been conducted to develop new bioactive and edible films from low-cost renewable natural resources and food industry wastes as alternatives to conventional packaging materials derived from limited and non-biodegradable resources [1,2,3]. One significant challenge is food waste, which is exacerbated by various factors throughout the supply chain, including spoilage and degradation during storage and transportation. Active packaging materials that help preserve food quality can play a critical role in addressing this issue [4]. This innovative and promising approach is an environmentally friendly technology that would minimize the accumulation of plastic products in natural surroundings, which adversely affects humans and wildlife and causes serious environmental problems [5,6]. Moreover, the application of bioactive films as packaging materials has attracted much attention in the food industry. Edible films and coatings have been involved to a large degree because of their ability to deliver active substances and their potential to improve food quality and safety, delay spoilage, maintain sensorial quality, and increase food shelf life [7,8].

Several potentially functional biopolymers, such as starch, cellulose, gluten, gelatin, chitosan and guar gum, have been widely studied to develop active packaging because of their attractive physicochemical and biological characteristics, good biodegradability and biocompatibility, renewability, non-toxicity and safety [2,5,9].

Chitosan is a natural-sourced polysaccharide obtained by deacetylation of chitin, consisting of β-(1→4)-linked d-glucosamine and N-acetyl-d-glucosamine units. Chitosan has recently become the focus of researchers and industrialists, as it exhibits several important advantages, such as renewability, biodegradability, non-toxicity, biocompatibility, film-forming properties, water retention ability and chelating/adsorption capacities [10,11,12]. Chitosan has received considerable attention in the development of active packaging because of its particular properties. Chitosan-based films are proven to decrease water loss, improve food texture and color stabilization, enhance natural flavor and delay enzymatic browning and microbial spoilage [13,14]. However, despite its many advantages, chitosan has notable limitations as a stand-alone film-forming material. Chitosan-based films often exhibit low mechanical strength, poor water resistance and limited barrier properties, particularly under humid conditions. These drawbacks restrict its application in active packaging and necessitate the inclusion of other biopolymers or additives to enhance its functional and structural properties [15,16].

Biopolymers derived from algae have also attracted particular attention as they are abundant in nature and are renewable and good sources of nutrients [17,18,19]. Although they have long been utilized in the food industry for phycocolloid extraction (carrageenan, agars and alginates) or as whole sea vegetables [20], nowadays, the use of algae as sustainable and safe alternative sources of medication and healthy food for the world’s expanding population is becoming increasingly popular [21]. *Ulva* and several other algae species are considered important functional foods, since they offer a wide range of natural bioactive compounds and metabolites (such as phlorotannins, sulfated polysaccharides (SPs), carotenoid pigments, phytosterols, fatty acids, polysaccharides, vitamins, minerals and bioactive peptides) which have a variety of biological activities, including anthelmintic, cytostatic, antiviral, antibacterial and antifungal properties [21,22,23] search on novel and diverse biobased materials made from algae is continuously being investigated. Algae-based films have gained significant attention because they showed excellent characteristics as they are biodegradable, biocompatible, non-toxic, impervious to fats and oils, have good optical and barrier properties and have high water-retaining capacity [24,25].

Edible films made from one type of biopolymer as packaging material present significant limitations, such as poor mechanical and barrier properties [5]. It was shown that the use of pure chitosan films has been restricted because of instability in the aqueous conditions and poor mechanical parameters, as well as moisture and gas barrier problems [26,27]. Moreover, previous studies report that agar film exhibited fragility and high permeability to water vapor [14]. Therefore, to overcome this drawback and to improve the physical and technofunctional properties of films, an alternative approach to mixing biopolymers into biocomposite materials is applied [28].

In recent years, numerous active systems have been created using algae extracts and chitosan, together or with other biopolymers, as functional compounds [29,30,31]. Various studies on seaweed/starch-, seaweed/cellulose- and seaweed/gelatin-based composites have been conducted, reporting excellent properties and good applicability in the food and pharmaceutical industries [5,32,33]. Moreover, several support polymers have been added to chitosan films (nanoparticles, tannic acid, polylactic acid, kojic acid, citric acid, metal ions, phenols, plant extract, cellulose, starch, etc.) in order to enhance their physicochemical and biological characteristics [26,33,34,35,36,37]. An edible film based on chitosan with alginate and fucoidan from brown macroalgae showed high antioxidant activities with increased thickness and decreased solubility [38]. Balti et al. [2] confirmed that edible films made from crab chitosan and enhanced with Spirulina extract exhibited significant potential for use in active food packaging owing to their outstanding antioxidant and antibacterial properties. Sinaga et al. [39] demonstrated that adding chitosan to agar enhances its mechanical properties due to cross-links created between molecules.

However, based on the vast literature, no studies were found showing the potential utilization of all of the *Ulva* algae components, with their functionalities and bioactivities, in the form of powder in combination with chitosan for a multifunctional (antioxidant and antimicrobial) active packaging system. Hence, this work aimed to develop a new sustainable, economical and environmentally friendly bioactive packaging system formulated with *Ulva lactuca* algae powder and crab (*Portunus segnis*) chitosan. With this aim, the physicochemical, mechanical, functional and biological properties of composite films were evaluated for potential applications in the food packaging industry.

## 2. Materials and Methods

### 2.1. Materials

Chitosan was extracted from blue crab (*Portunus segnis*) shells via chitin formation by chemical treatment according to the method of Zaghbib et al. [10]. Briefly, fresh crab shells were powdered (CSP) by a grinder (Retsch PM 100) after washing and drying at 40 °C. The demineralization process was carried out by treating the CSP with 1 M HCl (1:15, *w*/*v*) at 25 °C for 1 h. The obtained solid fraction was treated with 1 N NaOH (1:15, *w*/*v*) at 80 °C for 6 h for deproteinization. After decolorization with hydrogen peroxide solution (1:10, *w*/*v*), the obtained final material was termed chitosan.

Green algae *Ulva lactuca* was obtained from the Cap Zebib collecting station (North of Tunisia). After washing with tap water to remove impurities, algae samples were dried at room temperature until a constant weight. Finally, dried material was ground to make fine and homogeneous powder, as described by Zaghbib et al. [22].

The obtained powders were stored using a polyethylene bag and kept at room temperature in a desiccator for further use as active compounds.

Glycerol, acetic acid, 2,2-diphenyl-1-picrylhydrazyl (DPPH) and other chemical reagents were purchased from Sigma-Aldrich (Saint Louis, MO, USA).

All microorganisms used to assess the antimicrobial properties in this study were obtained from the culture collection of the Research Laboratory “Technological Innovation and Food Security”, Higher Institute of Food Industries of Tunisia, in nutrient agar and stored at 4 °C.

### 2.2. Composite Films Preparation via the Casting Method

#### 2.2.1. Preparation of Chitosan and Algae Coating Solutions

Chitosan and algae coating solutions were prepared following the method of Samani et al. [13], with slight modifications. The chitosan-film-forming solution was created by dispersing 1 g of the biopolymer in 1% (*v*/*v*) acetic acid. To prepare the algae solutions, *Ulva lactuca* powder (ULP) was dissolved in 100 mL of distilled water at varying concentrations (1%, 3%, and 5% *w*/*v*). Subsequently, 20% (*v*/*v*) glycerol (based on preliminary tests, was added separately to both the *Ulva lactuca* and chitosan solutions as a plasticizer based on their contents. The mixtures were continuously stirred and heated in a water bath at 90 °C and 60 °C, until dissolved. Finally, the resulting solutions were filtered through a gauze pad.

#### 2.2.2. Preparation of Composite Films

Biodegradable films were prepared using the casting solvent method, as described by Sinaga et al. [39], with minor modifications. Composite films were produced by blending chitosan and algae coating solutions in a 1:1 (*v*/*v*) ratio. Three different chitosan–algae powder films (CH-ULP Fs) were obtained at the following final concentrations of ULP (g/100 mL film solution) (Table 1): blend 1 (CH-ULP 0.5%), blend 2 (CH-ULP 1.5%), and blend 3 (CH-ULP 2.5%).

**Figure 1:**
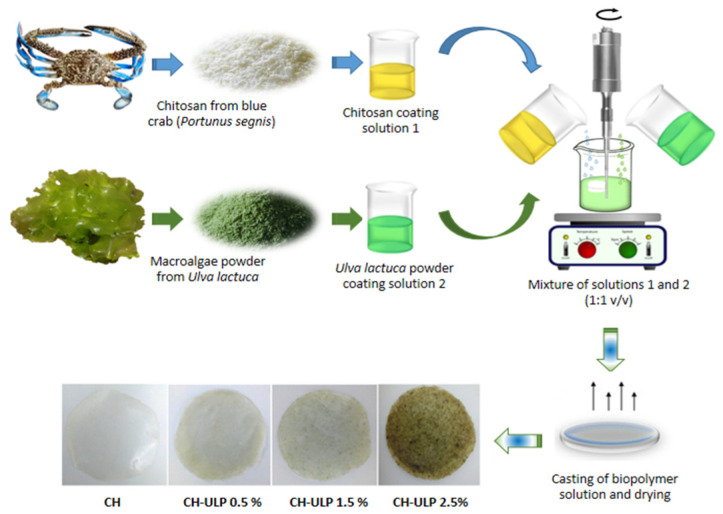
Preparation process of the chitosan–Ulva lactuca powder films. CH: chitosan-control film; CH-ULP 0.5%: Chitosan–algae film with 1% Ulva lactuca powder; CH-ULP 1.5%: Chitosan–algae film with 3% Ulva lactuca powder; CH-ULP 2.5%: Chitosan–ULP film with 5% Ulva lactuca powder.

The blended coatings were stirred with a homogenizer (Ultra-Turrax T25, IKA, Staufen, Germany) at 600 rpm for 60 min at 60 °C, until the mixtures became clear. Subsequently, CH-ULP Fs were prepared by casting 25 mL of the film-forming solutions into Petri dishes (13.5 cm in diameter) and drying them in an oven at 40 °C for 24 h. After drying, the films were removed from the dishes and placed in a temperature and humidity-controlled chamber set at 25 °C and 50% relative humidity (RH) for a minimum of 48 h prior to further analysis. A chitosan film (0% algae powder) was prepared as a control sample. The preparation process of the CH-ULP films is illustrated in Figure 1.

### 2.3. Characterization of Films

#### 2.3.1. Moisture Content

The moisture content of films was determined according to the method of Kumar et al. [40]. A total of 10 grams of each film sample was dried in an oven at 120 °C for 24 h. The moisture content, expressed as a percentage, was calculated according to the following formula:

Moisture (%) = [(W_0_ − W_1_)/W_0_] × 100 (1)

where W_0_ is the weight of the film before drying (g), and W_1_ is the weight of the film after drying (g).

#### 2.3.2. Water Solubility (WS)

The water solubility of the films was evaluated using the method of Najwa et al. [7]. To find the initial dry weight (W_0_), the film samples (discs: 16 cm^2^) were first dried in an oven for 24 h at 100 °C. The dried samples were then immersed in 30 mL of distilled water and left for 24 h at 25 °C under continuous stirring. After, samples were removed from the water, filtered through Whatman No. 1 filter paper and dried again (100 °C for 24 h) to obtain the final dry weight, W_1_. The edible films’ solubility (%) was calculated using the following equation:

Solubility (%) = [(W_0_ − W_1_)/W_0_] × 100 (2)

where W_0_ is the initial dry weight of the film (g), and W_1_ is the final dry weight of the film (g).

#### 2.3.3. Color

The color parameters of the films were determined using a Minolta colorimeter (CR 400; Minolta, Japan). The lightness (L*), redness (a*) and yellowness (b*) values of the developed films were measured by placing the films on the surface of a standard white plate. The total color difference (ΔE), whiteness index (WI) and yellowness index (YI) were determined according to Equations (3)–(5), as follows:

(3) $$\Delta E=\sqrt{{\left(\Delta L*\right)}^{2}+{\left(\Delta a*\right)}^{2}{+(\Delta b*)}^{2}}$$

(4) $$\mathrm{WI}=100-\sqrt{(100-{L*)}^{2}+{\left(a*\right)}^{2}+{\left(b*\right)}^{2}}$$

YI = 142.86 × (b*/L*)(5)

where ΔL*, Δa* and Δb* refer to the difference between the L*, a* and b* values of the films and those of the standard white plate (L*: 94.86; a*: −0.05; b*: 1.94).

#### 2.3.4. Opacity

The opacities of the films were determined according to the method of Liu et al. [26]. Edible films were cut in a rectangular shape, and the absorbance was measured at 600 nm using a UV spectrophotometer (Thermo Fisher Scientific, Madison, WI, USA). A blank (air) was used as the reference for comparative measurements. The following formula was used to calculate the film opacity:

Opacity = Abs_600_/T (6)

where Abs_600_ is the absorbance value at 600 nm; T is the film thickness (mm).

#### 2.3.5. Fourier Transform Infrared (FTIR) Spectroscopy

The Fourier transform infrared spectroscopy (FTIR) technique was used to determine the molecular structure and interactions among the functional groups of the different film samples using a Perkin Elmer spectrometer (Perkin Elmer, Waltham, MA, USA), as described by Najwa et al. [7] with slight modifications. The spectra were obtained between 4000 and 700 cm^−1^, averaging 32 scans with a resolution of 4 cm^−1^. The data were then analyzed using FTIR Spectrum software 10 (Perkin Elmer, Waltham, MA, USA).

#### 2.3.6. Mechanical Properties

The mechanical properties of the films were determined using an Instron Universal Testing Machine (Model 5655, Instron Corporation, Norwood, MA, USA), according to ASTM D1708-93 [41] and as described by Uranga et al. [7] with modifications. Samples with the same dimensions (10 mm × 25 mm) and thickness were stored at 23 °C with 50% relative humidity for at least 40 h. Then, film pieces were placed between the grip heads of the testing machine and stretched with the crosshead speed set at 1 mm/min and a load cell of 250 N. Tensile strength (TS) and elongation at break (EB) were determined after six measurements for each film.

#### 2.3.7. Antioxidant Activity: DPPH Radical Scavenging Assay

The antioxidant activities of the chitosan films incorporated with ULP were investigated using the DPPH radical scavenging assay. The DPPH radical scavenging effects of the control and blended coating solutions were estimated using the method of Butnaru et al. [42]. Film samples (300 mg) were immersed in 10 mL of chloroform for 24 h under continuous stirring to obtain the film extract solution. Then, a volume of 1.5 mL from each mixture was added to 3 mL of ethanolic DPPH solution (150 mM). The mixture was vigorously shaken and kept at room temperature in the dark for 30 min. A chloroform and DPPH solution without film extract was used as the control sample. The absorbance of the mixture was measured at 520 nm using a UV-Vis spectrometer (T70, UV/vis spectrometer, PG Instruments Ltd., Shenzhen, China), and the percentage of DPPH radical-scavenging activity was calculated according to the following equation:

DPPH scavenging effect (%) = [(A_control_ – A_sample_)/A_sample_] × 100 (7)

where A_sample_ is the absorbance of the sample solution in the presence of DPPH, and A_control_ is the absorbance of the standard DPPH solution.

#### 2.3.8. Antimicrobial Activity

The antimicrobial performances of the films were examined as the inhibitory effects against the growth of four bacteria (*Staphylococcus aureus* ATCC25923, *Listeria monocytogenes* ATCC 070101121, *Escherichia coli* ATCC2124 and *Salmonella typhimurium* ATCC 25922) and two fungi (*Geotrichum candidum* ATCC and *Aspergillus niger* ATCC) using the standard disc diffusion assay, as described by Zaghbib et al. [10] with slight modifications. To carry out the assay, a culture suspension of each microorganism (10^6^ cfu/mL) was spread on a Mueller–Hinton agar and potato dextrose agar for antibacterial and antifungal activities, respectively. Each biobased film was cut into a disc form (6 mm in diameter) and placed on the surface of the solid medium plates. Next, the plates were incubated for 24 h at 37 °C and 30 °C for bactericidal and fungicidal activities, respectively. In order to assess the antimicrobial activity, the diameters of the inhibition zones surrounding the film discs were measured against the test organisms. All tests were performed in triplicate.

#### 2.3.9. Biodegradability

To assess the biodegradability of the developed films, a soil burial degradation test was conducted following the procedure described by Alqahtani et al. [43] with minor modifications. Firstly, the developed films were cut into 4 × 4 cm^2^ pieces and dried in an oven at 60 °C until a constant weight. Then, the samples were buried in natural soil at a depth of 4 cm from the surface for 12 days. To keep the soil moist, water (20 mL) was sprayed daily. Every two days, the samples were removed from the soil, washed with distilled water several times, and dried (at 60 °C) until a constant weight. The biodegradability is expressed as the percentage of weight loss using the following equation:

Biodegradability (%) = [(W_0_ – W_1_)/W_0_] × 100 (8)

where W_0_ is the initial dry weight of the film sample (g), and W_1_ is the dry weight of the film sample after 12 days (g).

### 2.4. Statistical Analysis

Statistical analysis was performed using one-way analysis of variance (ANOVA) followed by Duncan’s multiple range tests to determine statistically significant differences among the sample sets. All analyses were conducted using SPSS software (SPSS Inc., Chicago, IL, USA, Version 25), with a significance level of *p* < 0.05. The results are reported as the mean ± standard deviation (SD) based on triplicate measurements. Different superscripts (e.g., a, b and c) indicate statistically significant differences between groups within tables and graphs. Where applicable, the coefficient of determination (R^2^) for the fitted models was also calculated and is reported to demonstrate the goodness of fit for the statistical analysis.

## 3. Results and Discussion

### 3.1. Moisture Content

The moisture content values for the various levels of *Ulva lactuca* powder incorporation into the chitosan–algae composite films are summarized in Table 2. The results indicate that the inclusion of *Ulva lactuca* powder significantly reduced the moisture content of the composite films compared to the chitosan control film (*p* < 0.05), with an *R*^2^ of 0.976. Additionally, a clear trend emerged, showing a significant decrease in moisture content as the concentration of *Ulva lactuca* powder increased, with the lowest moisture content observed at the 2.5% concentration level (*p* < 0.05). These findings are consistent with previous studies. For instance, Samani et al. [10] reported similar reductions in moisture content when agar from *Gracilaria corticata* was combined with chitosan in composite films. Similarly, Liu et al. [26] observed a marked decrease in moisture content when higher concentrations of kojic acid were incorporated into chitosan–kojic-acid composite films (CHn-KAm). The observed decrease in moisture content with an increasing *U. lactuca* powder concentration can be attributed to the molecular interactions within the film’s matrix. *U. lactuca* contains hydroxyl groups that can form hydrogen bonds with the amine groups in chitosan, reducing the availability of free hydrophilic groups (e.g., –OH groups) that would otherwise attract and retain water (for further information, refer to Section 3.5). This reduction in free hydrophilic groups likely limits the ability of the film to absorb water, leading to lower moisture content. Despite the overall reduction in moisture content with increased *U. lactuca* incorporation, the films still exhibited relatively high moisture contents (ranging from 13.6% to 19.7%). This can be attributed to the porous structure of the films and the storage conditions, particularly the high relative humidity, which could facilitate moisture absorption from the surrounding environment into the films’ structures. Jirukkakul [44] also highlighted that such factors significantly impact the moisture contents of biopolymer films.

### 3.2. Water Solubility (WS)

Water solubility (WS) is one of the key factors that restrict the potential use of biopolymer films as an alternative to petroleum-based plastics, as it influences the film’s integrity, biodegradability, and resistance in aqueous conditions [31,43]. The WS values of the developed-based films ranged from 15.4 ± 0.02 to 24.4 ± 0.03% (Table 2). Samples made from chitosan and *Ulva lactuca* powder exhibited significantly lower WS values (*p* < 0.05) than the control, which was made from chitosan only (27.9 ± 0.02%). The WS of CH-ULP films showed a linear decrease with an augmented amount of ULP (*p* < 0.05), with an *R*^2^ value of 0.995, consistent with the moisture content results. The film’s solubility at 2.5% incorporation level (15.4 ± 0.02%) was significantly lower (*p* < 0.05) than for other treatments. Similar findings were found by Govindaswamy et al. [45], who observed a decrease in the water solubility of biocomposite films developed with extracted fish (*Labeo rohita*) gelatin and fucoidan from brown seaweed (*Padina tetrastromatica*). Kumar et al. [40] reported that the solubility of ordinary chitosan film was higher compared to films containing pomegranate peel extract (PPE), with the solubility decreasing as the concentration of PPE increased. Previous researchers have also reported a decreasing trend in solubility after the incorporation of seaweed and Ginkgo biloba extract in alginate and gelatin films, respectively [46,47]. In contrast to this work, Gomaa et al. [38] used ulvan extracts, which are a water-soluble sulphated polysaccharide and the main component of *Ulva* species, to develop cellulose–ulvan biocomposite films. They report that the WS of the biocomposite films were significantly higher compared to cellulose film because of the small molecular weight and the hydrophilic nature of ulvan. However, increasing the ulvan concentration from the green seaweed *Ulva lactuca* in the developed films had no significant impact on the WS values. According to Don et al. [48], the swelling degree was the lowest for the chitosan film, and it increased three-fold for the ulvan/chitosan/tripolyphosphate complex films. The decreases in the WS films could be explained by a decrease in interactions between CH-ULP and water molecules. Since *Ulva lactuca* is considered a potentially rich source of natural antioxidants [21,23] and, thus, contains a significant amount of phenolic compounds, which may increase the hydrophobicity and the strengthening of the polymer network in the developed films [40]. In the formed CH-ULP film, interactions between carboxylic groups and phenolic hydroxyls may occur, limiting the interaction with water and creating more resistant films (for further information, refer to Section 3.5). Food coatings must have high solubility to be easily consumed and digested by the body to release essential substances. However, low-dissolving films are commonly used in food packaging to maintain the integrity of food structures [38,44].

### 3.3. Visual Observations and Color Measurements

**Figure 2:**
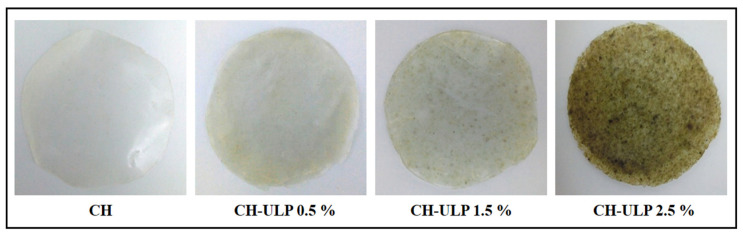
Color changes in the developed films. CH: chitosan-control film; CH-ULP 0.5%: Chitosan–algae film with 1% *Ulva lactuca* powder; CH-ULP 1.5%: Chitosan–algae film with 3% *Ulva lactuca* powder; CH-ULP 2.5%: Chitosan–ULP film with 5% *Ulva lactuca* powder.

Color is an important characteristic of films, determining the first aspect of food products influencing consumer acceptance, consideration, and willingness to buy a product [49]. Figure 2 displays the visual appearance of the chitosan–algae composite films, revealing clear morphological and structural differences across samples. The control chitosan film (CH) exhibited a smooth, transparent and uniform surface, characteristic of pure chitosan. However, the addition of *Ulva lactuca* powder (ULP) induced concentration-dependent changes to the film’s surface. At 0.5% ULP, the film retained a relatively smooth and homogeneous appearance, with a slight yellow–green tint reflecting good dispersion of ULP particles. In contrast, CH-ULP 1.5% and CH-ULP 2.5% films showed more pronounced changes, including darker coloration, reduced transparency, and visible aggregates. These surface irregularities are attributed to the expulsion of ULP particles from the chitosan network, leading to aggregation on the film’s surface. The heterogeneous appearance observed at higher ULP concentrations is influenced by several factors, including the particle size, dispersion efficiency, granulation, increased viscosity of the film-forming solution, limited solubility of some ULP components and aggregation/re-aggregation during the solvent evaporation process [50]. Additionally, the hydrophobic nature of ULP compounds may contribute to aggregation, consistent with the reduced moisture content and solubility observed in Section 3.1 and Section 3.2. The reduction in molecules available for interaction with water likely promotes the clustering of nonpolar solutes in the solvent mixture, further exacerbating surface unevenness. Advanced dispersion techniques, such as ultrasonication, precise filtration, or surfactant incorporation, could improve the uniformity of the films, and further investigation using techniques like SEM or confocal microscopy can provide deeper insight into the surface morphology and particle distribution.

The incorporation of ULP into crab chitosan film significantly affected the color parameters of the film’s surface (*p* < 0.05) (Figure 2 and Table 3). The results show a decrease in the lightness (L*) (*R*^2^ = 0.777), redness (a*) (*R*^2^ = 0.476) and whiteness index (WI) (*R*^2^ = 0.897) values and an increase in the yellowness (b*) (*R*^2^ = 0.887), total color difference (ΔE) (*R*^2^ = 0.95) and yellowness index (YI) (*R*^2^ = 0.828) values compared to those of the chitosan film (*p* < 0.05). The L*, a* and WI values were higher in the crab chitosan film and those of the bio-blend films decreased significantly (*p* < 0.05) from 88.7 ± 0.16 to 85.7 ± 0.03, from −0.8 ± 0.03 to −1.1 ± 0.01 and from 88.6 ± 1.45 to 85.5 ± 0.45, respectively, when the ULP concentration increased from 0.5 to 2.5%.

The decrease in the L* values indicates film darkening and a reduction in transparency [46]. Because of this, the total color difference (ΔE) of the films increased with the addition of ULP, indicating increased coloration. The b* and YI values increased significantly (*p* < 0.05) with the addition of ULP from 1.1 ± 0.01 to 1.8 ± 0.06 and from 1.7 ± 0.45 to 3.0 ± 0.38 for the control and CH-ULP 2.5%, respectively, indicating the tendency of the films to exhibit green and yellow coloration in comparison to the control. Our results are in harmony with those reported by Samani et al. [13], who found that adding agar extracted from *Gracilaria corticata* macroalgae to the chitosan film and fucoidan extracted from *Sargassum angustifolium* macroalgae to the chitosan–agar film reduced the L*, a* and WI values while b* and ΔE values increased significantly. Govindaswamy et al. [45] also noted that the addition of fucoidan extracted from brown seaweed (*Padina tetrastromatica*) to gelatin-based film modified the color parameters resulting in reduced the lightness (L*) and whiteness (WI) values and increased the yellowness (b*) and total color difference (ΔE) values. Rattaya et al. [51] reported that incorporating seaweed extract into the gelatin films caused them to appear more yellow and green, leading to a significant decrease in the lightness (L*) and redness (a*) values compared to the control film. The results of this study show that the incorporation of ULP significantly affected the color properties of the resulting crab chitosan bio-blended films and led to a higher degree of darkening and enhanced yellow and green attributes. It has been reported that the color of a film is directly affected by the type of extract, concentration, and internal structure developed during film drying [8,26]. Balti et al. [2] attributed the difference in the color of chitosan–Spirulina extract films to the presence of antioxidant agents and colored substances in the seaweed. Opaque packaging completely blocks visible light, offering effective protection for products against photooxidation [52]. Opaque green and brown-colored films are commonly used for light-sensitive products, such as dairy items, pharmaceuticals, edible oils and beer [49,52]. These films are particularly beneficial for preserving the quality of products sensitive to light exposure.

### 3.4. Opacity

Opacity is an important physical property of films used for food packaging and coating, as transparency enables consumers to see the appealing appearance of a product before making a purchase. On the other hand, packaging material should also protect food from the effects of light, particularly UV radiation [45]. The opacity results of the developed films are presented in Table 3. The addition of various ULP concentrations (0.5–2.5%) significantly (*p* < 0.05) increased the opacity of the crab CH-ULP biocomposite films (*R*^2^ = 0.784). This observation is consistent with the color parameter results, which show that incorporating more ULP led to a darker color of the chitosan–ULP films. Gelatin film incorporated with 6% seaweed extract showed higher opacity values [51]. Similarly, the incorporation of fucoidan extracted from brown seaweed (*Padina tetrastromatica*) into fish gelatin film increased the opacity [45]. The increase in the film’s opacity could be due to the fact that *Ulva lactuca* exhibited high protein content and antioxidant compounds, as well as a light scattering effect [7,8,45]. According to a study by Zaghbib et al. [22], this macroalgae species contains 10.63 ± 0.2% (DW) of proteins and demonstrates a relatively high antioxidant capacity, at a 54.46 ± 0.39% DPPH radical inhibition at a concentration of 1000 μg/mL and a low IC_50_ value (896.77 ± 0.31 μg/mL). It is well known that antioxidant compounds have the capability to absorb UV light and, consequently, protect food against this type of radiation [31,38,53]. Generally, protein films containing high amounts of aromatic amino acids exhibit excellent UV barrier properties [45]. These results suggest that CH-ULP films may have interesting UV barrier properties. According to Gomaa et al. [38], the cellulose–ulvan (*Ulva lactuca*) film demonstrated low light transmittance at 280 nm and 400 nm wavelengths, indicating ulvan’s ability to block UV light effectively. Additionally, they report that at 280 nm, the film’s opacity markedly increased with higher ulvan concentration (*p* < 0.05), which confirms ulvan’s excellent UV barrier properties in the composite films.

### 3.5. Fourier-Transform Infrared (FTIR) Spectroscopy

**Figure 3:**
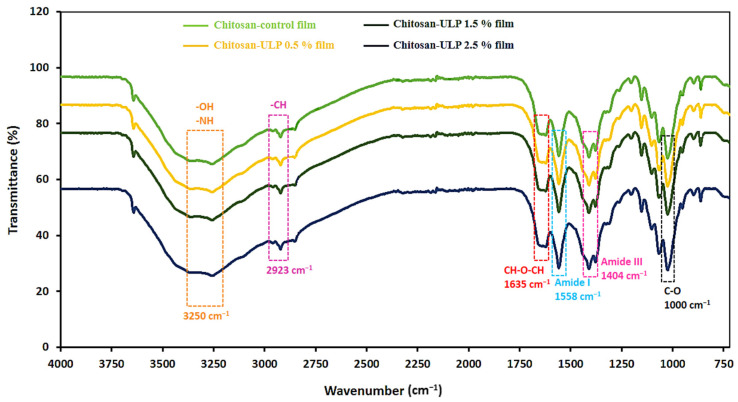
FTIR spectra of the chitosan–algae composite films. Chitosan-control film: chitosan film with 0% *Ulva lactuca* powder; Chitosan–ULP 0.5% film: chitosan–algae film with 1% *Ulva lactuca* powder; Chitosan–ULP 1.5% film: chitosan–algae film with 3% *Ulva lactuca* powder; Chitosan–ULP 2.5% film: chitosan–algae film with 5% *Ulva lactuca* powder.

In order to assess the molecular interactions among biopolymers, FTIR spectroscopy was carried out (Figure 3). The FTIR spectra of the chitosan films incorporated with various concentrations (0–2.5%) of ULP displayed similar patterns, with most of the bonds showing characteristics of chitosan film but with different transmittance intensities. The chitosan FTIR spectrum exhibited vibrations at 3250 cm^−1^ attributed to the hydroxyl (–OH) and amine (–NH) functional groups. Additionally, a small sharp bond at around 3600 cm^−1^, which corresponds to the stretching vibrations of free –OH groups, was present but less prominent compared to the broader bond at 3250 cm^−1^. The region from 3100 to 3700 cm^−1^ reflects the stretching vibrations of both intermolecular and intramolecular bonded –OH groups, as well as N–H amines [54,55]. The absorption bonds at 2923 cm^−1^, 1635 cm^−1^, 1558 cm^−1^, 1404 cm^−1^ and 1000 cm^−1^ can be attributed to –CH stretching, CH–O–CH stretching and N–H stretching vibrations of amide I and amide III and C–O bending vibration, respectively [2,14]. The FTIR spectra of the CH-ULP films are similar to the CH-film. The most significant change in the aforementioned bands can be associated with the decrease in band amplitude corresponding to the –OH and –NH stretching in the CH-ULP 1.5% and CH-ULP 2.5% films. This decrease indicates that the hydroxyl (–OH) and amine (–NH) functional groups of chitosan are involved in hydrogen bonding and possible covalent interactions with the polyphenolic compounds of ULP. These interactions reduce the availability of free hydroxyl and amino groups, which otherwise contribute to hydrophilic behavior. Moreover, the amide I (around 1635 cm⁻^1^) and amide III (around 1558 cm⁻^1^) bands are primarily derived from chitosan due to residual acetyl groups contributing to the N–H bending and C=O stretching vibrations. Although chitosan is predominantly a polysaccharide, incomplete deacetylation leaves functional groups that generate these bands. The addition of ULP amplifies the relative intensity of these bands, as polyphenolic compounds in ULP form hydrogen bonds and covalent interactions with chitosan’s amino and hydroxyl groups, stabilizing these signals. ULP, being rich in polyphenols and devoid of protein, does not inherently contribute to amide bands but modifies their intensity through molecular interactions with chitosan. Additionally, with the incorporation of ULP into CH film, the single band at around 1000 cm^−1^ in the chitosan control film became broader and divided into two overlapping bands in the films with ULP. These findings are consistent with previous studies that outlined the creation of bonds between the propolis–chitosan [56], chitosan matrix, and functional groups of polyphenolic compounds from spirulina extract [2]. These results suggest that intermolecular interactions occur between the functional groups of the polyphenolic compounds of ULP and the hydroxyl and amino groups of the chitosan matrix. Polyphenolic compounds from *Ulva lactuca* powder can form hydrogen and covalent bonds, thereby occupying the functional groups of the chitosan matrix, reducing the number of free hydrogen groups that would otherwise form hydrophilic bonds with water. In addition, these findings are in accordance with the previously stated results, as follows: (1) water content, where the reductions in the biocomposite films’ moisture contents after ULP addition were attributed to molecular interactions that occurred between the hydroxyl groups of the ULP and the amine groups of the chitosan; (2) water solubility, which decreased significantly (*p* < 0.05) with an augmented amount of ULP because of the establishment of carboxylic-phenolic hydroxyls interactions; and (3) color and (4) opacity, which were affected by the internal structures and interactions in the biocomposite CH-ULP films leading to a higher dark, yellow and green attributes.

### 3.6. Mechanical Properties

The mechanical properties are highly important for a material to be used in the packaging industry. A biodegradable membrane needs to have sufficient mechanical strength, flexibility, and extensibility to ensure the integrity and barrier properties of films proposed for food-packaging applications [8,26]. The effect of incorporating ULP into the chitosan–biopolymer matrix on the mechanical behavior of edible CH-ULP biocomposite films was investigated with regard to the elongation at break (EB) and tensile strength (TS) (Table 4). The mechanical testing results show that the chitosan control film exhibited TS and EB values of 33.4 ± 0.56 MPa and 3.0 ± 0.82%, respectively. The incorporation of ULP improved the mechanical properties of the films, since both TS and EB significantly (*p* < 0.05) increased from 41.4 ± 0.78 MPa (0.5% ULP) to 49.8 ± 0.53 MPa (2.5% ULP) (*R*^2^ = 0.966) and from 4.9 ± 0.54% (0.5% ULP) to 7.5 ± 1.37% (2.5% ULP) (*R*^2^ = 0.971), respectively. These results align with those reported by Najwa et al. [7], who stated that incorporation of *Garcinia atroviridis* leaf extracts to the gelatin-starch films improved the mechanical properties. Similarly, a previous study reported that adding pomegranate peel extract significantly enhanced the tensile strength of chitosan film because of the interaction between the matrix and phenolic fractions [8]. In addition, Balti et al. [2] found that incorporating spirulina extract significantly improved the mechanical strength of crab chitosan film due to the interactions between the matrix and the bioactive compounds of the seaweed extract. This is also in accordance with another study, which reported that the enhancement could be attributed to the interactions that occurred by hydrogen bonding between the extracts and the biopolymers [57]. The composition and the structure of materials used to develop films are related to the improvement in mechanical properties, especially those containing phenolic compounds and aromatic structures that provide films with higher tensile strength [7]. In this study, the compatibility of both the film-forming materials in terms of mechanical strength showed excellent results in a 1:1 combination of chitosan and *Ulva lactuca* polymers. The improvement in the mechanical properties could be related to the uniform and homogeneous distribution of ULP particles within the chitosan matrix. Moreover, intermolecular interactions characterized by hydrogen bonding between the amine group of the chitosan and the hydroxyl groups of the phenolic compounds present in the ULP could be responsible for the mechanical properties’ enhancement, which was demonstrated previously by FTIR analysis. Thus, the natural and phenolic compounds of ULP provide rigidity to a film by acting as a natural filler that reinforces the chitosan matrix.

### 3.7. Antioxidant Activity

The ability of packaging film compounds to act as free-radical scavengers or hydrogen donors and to inhibit or reduce oxidation is of great importance for packaged foods and human health. Moreover, active coatings and films with antioxidant properties may influence the direct addition of antioxidants into foods, help to reduce and control disease risks, increase the stability of packaged food products and extend their shelf life. Antioxidant properties are fundamental, as they benefit many other properties of active films, including extending the functional shelf life of packaged products, preventing oxidative deterioration, and enhancing the overall protective functionality of the packaging [58,59].

**Figure 4:**
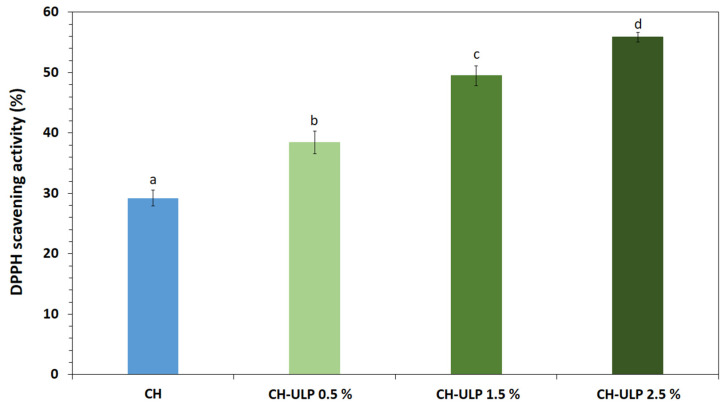
Radical scavenging activity of chitosan–algae composite films. CH: Chitosan-control film; CH-ULP 0.5%: Chitosan–algae film with 1% *Ulva lactuca* powder; CH-ULP 1.5%: Chitosan–algae film with 3% *Ulva lactuca* powder; CH-ULP 2.5%: Chitosan–algae film with 5% *Ulva lactuca* powder. Values are given as mean ± SD. Different lowercase letters (a–d) indicate significant differences (*p* < 0.05) based on Duncan’s test.

In this study, the DPPH free-radical scavenging assay was chosen because of its simplicity, reliability, and widespread use as a primary method for evaluating antioxidant activity. The DPPH technique specifically measures the ability of antioxidants to donate hydrogen atoms or electrons to neutralize free radicals, providing a direct indicator of the radical scavenging potential of the film components [60]. While this study employed only one method, the results are consistent with previous findings and highlight the significance of the polyphenolic compounds in Ulva lactuca powder (ULP). Future studies will incorporate additional techniques, such as ferric-reducing antioxidant power (FRAP) and ABTS assays, to further elucidate the antioxidant mechanisms and activity profiles of the films. As shown in Figure 4, the 100% crab chitosan-based film showed the lowest scavenging activity on DPPH (29.2 ± 1.33%), thus holding some antioxidant activity. With the increase in the ULP concentration added to the chitosan film, the DPPH-radical scavenging capacity significantly increased (*p* < 0.05) (*R*^2^ = 0.989). Samples formulated with ULP 2.5% exhibited the highest activity (55.9 ± 0.76%). These findings are in accordance with previous studies that reported an increase in DPPH-radical scavenging activity of chitosan films incorporated with spirulina extracts [2], *Himanthalia elongata* and *Palmaria palmata* seaweeds [61], agar and fucoidan extracted from *Gracilaria corticata* and *Sargassum angustifolium* macroalgae [14]. Furthermore, the antioxidant capacity of *Ulva* species has been proved by previous studies [22,38]. It has been reported that ulvan exhibits antioxidant properties. In fact, the strong hydrogen supply ability, metal-chelating activity and reducing power of ulvan are attributed to its glucuronic acid, hydroxyl groups and sulfate groups [38,48]. These findings highlight the potential of this macroalgae species to act as a natural antioxidant.

### 3.8. Antimicrobial Activity

**Figure 5:**
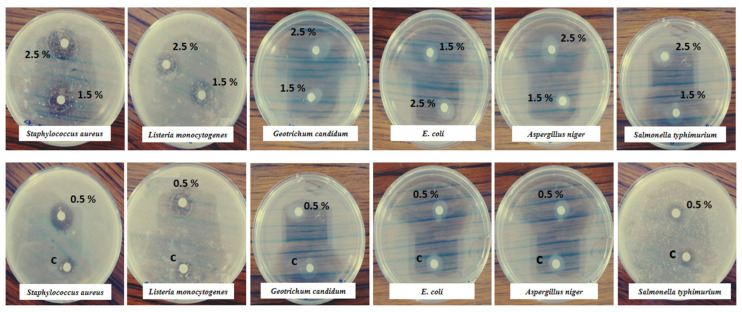
Visible zone of inhibition produced by 1, 3 and 5% *Ulva lactuca* powder incorporated chitosan-edible film. C: Chitosan-control film; 0.5%: Chitosan–algae film with 1% *Ulva lactuca* powder; 1.5%: Chitosan–algae film with 3% *Ulva lactuca* powder; 2.5%: Chitosan–algae film with 5% *Ulva lactuca* powder against *Staphylococcus aureus*, *E. coli*, *Salmonella typhimurium*, *Listeria monocytogenes*, *Geotrichum candidum* and *Aspergillus niger* by the disc diffusion method.

Food active packaging needs to demonstrate antimicrobial activity, as microbial activity is one of the primary causes of food spoiling. The results of the antimicrobial effects of the CH, CH-ULP 0.5%, CH-ULP 1.5% and CH-ULP 2.5% films against four bacteria and two fungi are presented in Table 5 and Figure 5. The results reveal that both the CH-control and CH-ULP films exhibited antimicrobial activity against all tested microorganisms. These findings are in accordance with Zaghbib et al. [10,22], who stated that chitosan and ULP were effective against various microorganisms (*Escherichia coli*, *Salmonella typhimurium*, *Staphylococcus aureus*, *Listeria monocytogenes*, *Aspergillus niger* and *Geotrichum candidum*) and inhibited them effectively. However, Balti et al. [2] and Wang et al. [62] reported that no significant inhibition zone was observed for a pure chitosan film against *E. coli*, *P. aeruginosa*, *L. monocytogenes*, *S. typhimurium*, *B. subtilis*, *B. cereus* and *S. aureus*. According to previous studies, *Ulva lactuca* and green algae exhibited greater activity than other groups of algae evaluated for their antibacterial activity [63]. The chitosan edible films incorporated with ULP showed a significant (*p* < 0.05) antimicrobial activity against fungi and Gram-positive and Gram-negative bacteria. The diameters of the inhibition zones of chitosan-based composite films incorporated with ULP from 0.5% to 2.5% were 12.4 ± 0.02 to 17.0 ± 0.02 mm against *Escherichia coli* (*R*^2^ = 0.988); 12.0 ± 0.04–15.1 ± 0.03 mm against *Salmonella typhimurium* (*R*^2^ = 0.96); 18.2 ± 0.03 to 24.0 ± 0.08 mm against *Staphylococcus aureus* (*R*^2^ = 0.842); 16.9 ± 0.08 to 23.2 ± 0.04 mm against *Listeria monocytogenes* (*R*^2^ = 0.785); 13.7 ± 0.01 to 16.1 ± 0.13 mm against *Aspergillus niger* (*R^2^* = 0.951); and 14.2 ± 0.01 to 17.1 ± 0.02 mm against *Geotrichum candidum* (*R*^2^ = 0.91). These edible films showed an inhibitory effect that was concentration-dependent. Obviously, the chitosan-based composite films with varied amounts of ULP demonstrated strong bacteriostatic properties against all bacterial strains tested, as the inhibition zone increased with the addition of more ULP (Figure 5). The highest potency of ULP against bacteria was detected toward *Staphylococcus aureus* (24.0 ± 0.08) and *Escherichia coli* (17.0 ± 0.06 mm) for Gram-positive and Gram-negative strains, respectively, at a concentration of 2.5%. On the other hand, *Geotrichum candidum* showed the highest inhibition halos (17.1 ± 0.02 mm) at a concentration of 2.5%. Our results suggest that the addition of ULP improved the antimicrobial activity of the chitosan films and was more effective against Gram-positive bacteria (*Staphylococcus aureus*) than Gram-negative bacteria and fungi. These findings are in harmony with those reported by Balti et al. [2], who stated that edible chitosan films incorporated with spirulina extracts had an inhibitory effect, at all concentrations, on five bacteria assayed (*E. coli*, *S. aureus*, *L. monocytogenes*, *B. subtilis* and *B. cereus*). According to Liu et al. [26], the antibacterial activity of the films may be the result of various properties, including changes in membrane permeability, impaired nucleic acid synthesis, and chemical changes in microbial organelles, enzymes, or proteins, ultimately leading to microbial cell death. Gram-positive bacteria are usually regarded as more sensitive than Gram-negative bacteria to antimicrobial compounds [2]. This could be due to structural differences in their cell walls, as the cell walls of Gram-negative bacteria contain lipopolysaccharides that may prevent active components from accessing the cytoplasmic membrane [2].

### 3.9. Biodegradability

**Figure 6:**
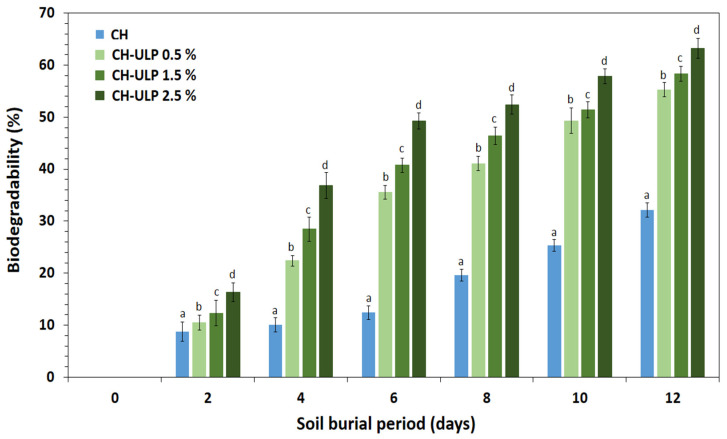
Biodegradability (%) of chitosan–algae composite films over 12 days. CH-ULP 0.5%: Chitosan–algae film with 1% *Ulva lactuca* powder; CH-ULP 1.5%: Chitosan–algae film with 3% *Ulva lactuca* powder; CH-ULP 2.5%: Chitosan–algae film with 5% *Ulva lactuca* powder. Different lowercase letters (a–d) for the same day are significantly different (*p* < 0.05) based on Duncan’s test.

Biodegradability is the process of breaking down organic matter by microorganisms present in the soil [64]. The results of the biodegradability test are presented in Figure 6. After six days of burial, the percentages of biodegradability were 12.4 ± 1.27, 35.56 ± 1.35, 40.8 ± 1.36, 49.3 ± 1.54% for CH, CH-ULP 0.5%, CH-ULP 1.5%, and CH-ULP 2.5%. After 12 days of burial in compost soil, the films showed perfect decomposition results as an indicator of biodegradability, with the best value of film weight loss observed for the sample developed with 5% ULP (63.3 ± 1.88%). The results obtained exceed the minimum biodegradation limit, with the threshold set at 60% for a period of 10 to 28 days [64]. These findings are in accordance with those reported by Alqahtani et al. [43], who reported that the weight of the films developed from corn starch and date palm pits (*Phoenix dactylifera*) dramatically decreased after 10 days of burial in soil. The components used in this study for the development of biobased films were isolated from living organisms (algae and crab), which are biodegradable and, thus, could be directly integrated into the soil, where the microorganisms convert them into water, carbon dioxide or methane, and biomass [65]. The film degradation process is related to several physical, chemical and biological factors, such as soil moisture, chemical structure, melting point, molecular weight, surface area, hydrophilic and hydrophobic properties and disintegration caused by the microorganisms present in the soil [43,64]. The developed films represent an ecologically sustainable alternative to conventional films. In fact, this type of biobased film can be considered biodegradable, biocompatible and bioactive.

## 4. Conclusions

The present study involved the successful fabrication of chitosan-based active films incorporated with various concentrations of *Ulva lactuca* powder. The incorporation of ULP at various concentrations into the chitosan films significantly increased the opacity, while the moisture content and water solubility significantly decreased. The films, analyzed by FTIR, showed good chemical interaction between the chitosan and ULP, forming a stable composite film with excellent integrity. The composite films were also characterized by enhanced mechanical properties, which led to stronger and more flexible films. Improvements in the barrier properties against UV-Vis light were concluded because of the increased opacity and color due to the strong interaction of polyphenols within the polymer matrix. In addition, all investigated films displayed potential antioxidant and antimicrobial properties, making them ideal choices for environmentally friendly food packaging material with improved biological properties that can enhance food safety and extend the shelf life of perishable foods. However, biodegradable films must be guaranteed to be free of heavy metals, which can pose risks to human health and the environment. Additional studies are necessary to explore the impact of incorporating ULP into chitosan film on the heavy metal content.

## Figures and Tables

**Table 1:** Composition of the developed films.

Blend	Chitosan (%, *w*/*v*)	ULP (%, *w*/*v*)	Glycerol (%, *v*/*v*)	Ratio (*v*/*v*)
Control: CH	1	0	20	-
Blend 1: CH-ULP 0.5%	1	1	20	1:1
Blend 2: CH-ULP 1.5%	1	3	20	1:1
Blend 3: CH-ULP 3%	1	5	20	1:1

The table presents the compositions of the developed films. CH represents the control (chitosan only), and the CH-ULP blends include varying concentrations of algae composite (ULP) at 0.5%, 1.5%, and 3% (*w*/*v*), with a consistent chitosan concentration (1%, *w*/*v*) and glycerol as the plasticizer (20%, *v*/*v*) at a fixed ratio of 1:1 (*v*/*v*).

**Table 2:** Moisture contents (%) and solubilities (%) of the chitosan–algae composite films.

Biobased Film	Moisture Content	Solubility
CH	22.3 ± 0.01 ^a^	27.9 ± 0.02 ^a^
CH-ULP 0.5%	19.7 ± 0.01 ^b^	24.4 ± 0.03 ^b^
CH-ULP 1.5%	17.7 ± 0.03 ^c^	20.1 ± 0.01 ^c^
CH-ULP 3%	13.6 ± 0.02 ^d^	15.4 ± 0.02 ^d^

Values are expressed as the mean ± standard deviation (SD) of triplicate measurements. Within the same column, values followed by different superscripts (^a–d^) are significantly different (*p* < 0.05) when analyzed by Duncan’s test, indicating variations among the different compositions of chitosan–algae composite films (CH: chitosan only; CH-ULP: chitosan with varying concentrations of algae composite of 0.5%, 1.5%, and 3%).

**Table 3:** Color parameters (L*, a* and b*), color difference (ΔE), whiteness index (WI), yellowness index (YI) and opacity of chitosan–algae composite films.

Parameters	Biobased Films			
	CH	CH-ULP 0.5%	CH-ULP 1.5%	CH-ULP 3%
L*	89.4 ± 0.04 ^a^	88.7 ± 0.16 ^a^	86.6 ± 0.02 ^b^	85.7 ± 0.03 ^c^
a*	−0.8 ± 0.03 ^a^	−0.8 ± 0.03 ^a^	−0.9 ± 0.02 ^b^	−1.1 ± 0.01 ^c^
b*	1.1 ± 0.01 ^a^	1.2 ± 0.02 ^a^	1.4 ± 0.05 ^b^	1.8 ± 0.06 ^c^
ΔE	5.5 ± 1.23 ^a^	6.2 ± 0.58 ^a^	8.3 ± 1.87 ^b^	9.4 ± 0.77 ^c^
WI	89.5 ± 1.34 ^a^	88.6 ± 1.45 ^a^	86.5 ± 0.07 ^b^	85.5 ± 0.45 ^c^
YI	1.7 ± 0.45 ^a^	1.9 ± 0.28 ^a^	2.2 ± 1.83 ^b^	3.0 ± 0.38 ^c^
Opacity (%)	16.8 ± 1.43 ^a^	19.3 ± 0.07 ^b^	19.8 ± 0.28 ^b^	20.3 ± 0.75 ^c^

Values are expressed as the mean ± standard deviation (SD) of triplicate measurements. Within the same row, values followed by different superscripts (^a–c^) are significantly different (*p* < 0.05) when analyzed by Duncan’s Test, indicating variations among the different compositions of chitosan–algae composite films (CH: chitosan only; CH-ULP: chitosan with varying concentrations of algae composite of 0.5%, 1.5%, and 3%).

**Table 4:** Mechanical properties (tensile strength (TS) (MPa) and elongation at break (EB) (%)) of the chitosan–algae composite films.

Biobased Film	Tensile Strength (MPa)	Elongation at Break (%)
CH	33.4 ± 0.56 ^a^	3.0 ± 0.82 ^a^
CH-ULP 0.5%	41.4 ± 0.78 ^b^	4.9 ± 0.54 ^b^
CH-ULP 1.5%	44.2 ± 1.03 ^c^	5.7 ± 0.24 ^c^
CH-ULP 3%	49.8 ±0.53 ^d^	7.5 ± 1.37 ^d^

Values represent the mechanical properties of the chitosan–algae composite films, expressed as the mean ± standard deviation (SD) of triplicate measurements. The tensile strength (TS) is expressed in megapascals (MPa) and elongation at break (EB) as a percentage (%). Within the same column, values followed by different superscripts (^a–d^) are significantly different (*p* < 0.05), indicating variations in the mechanical properties among the different compositions of chitosan–algae composite films (CH: chitosan only; CH-ULP: chitosan with varying concentrations of algae composite at 0.5%, 1.5% and 3%).

**Table 5:** Antimicrobial effects of chitosan–algae composite films against pathogenic microorganisms determined via the disc diffusion method.

Pathogenic Microorganism	Diameter of Inhibition Zone (mm)			
	CH	CH-ULP 0.5%	CH-ULP 1.5%	CH-ULP 3%
*Escherichia coli*	10.0 ± 0.06 ^a^	12.4 ± 0.02 ^b^	14.1 ± 0.01 ^c^	17.0 ± 0.06 ^d^
*Salmonella typhimurium*	11.1 ± 0.03 ^a^	12.0 ± 0.04 ^b^	13.1 ± 0.04 ^c^	15.1 ± 0.03 ^d^
*Staphylococcus aureus*	16.1 ± 0.08 ^a^	18.2 ± 0.03 ^b^	18.5 ± 0.01 ^c^	24.0 ± 0.08 ^d^
*Listeria monocytogenes*	15.3 ± 0.04 ^a^	16.9 ± 0.08 ^b^	17.1 ± 0.09 ^c^	23.2 ± 0.04 ^d^
*Aspergillus niger*	12.1 ± 0.13 ^a^	13.7 ± 0.01 ^b^	14.2 ± 0.02 ^c^	16.1 ± 0.13 ^d^
*Geotrichum candidum*	14.1 ± 0.02 ^a^	14.2 ± 0.01 ^a^	16.1 ± 0.01 ^b^	17.1 ± 0.02 ^c^

Values represent the diameter of the inhibition zone (mm) measured via the disc diffusion method. The results are expressed as the mean ± standard deviation (SD) of triplicate measurements. Within the same row, values followed by different superscripts (^a–d^) are significantly different (*p* < 0.05), indicating variations in antimicrobial activity among the different concentrations of chitosan–algae composite films (CH: chitosan only; CH-ULP: chitosan with varying concentrations of algae composite at 0.5%, 1.5% and 3%).

## Data Availability

The original contributions presented in this study are included in the article. Further inquiries can be directed to the corresponding authors.
